# How I Teach It: Building an Extracorporeal Membrane Oxygenation and Mechanical Circulatory Support Fellowship for General Surgery Residents

**DOI:** 10.1016/j.atssr.2025.12.004

**Published:** 2025-12-24

**Authors:** Elizabeth J. Bashian, Thomas F. O’Shea, Sarah Y. Park, Nicholas R. Teman, Jordan R.H. Hoffman, Michael T. Cain

**Affiliations:** Division of Cardiothoracic Surgery, University of Colorado Anschutz Medical Campus, Aurora, Colorado

Since the early 2000s, improvements in extracorporeal membrane oxygenation (ECMO) design and management have led to an increase in ECMO use, with duration extended from days to weeks and expansion of indications, including bridge to heart or lung transplant and periprocedural support for cardiopulmonary interventions.^1^, ^2^, ^3^, ^4^ Despite this expanded use of ECMO, training or credentialing for ECMO remains inconsistent. Most ECMO cannulations are performed by cardiothoracic surgeons, but they are increasingly being performed by other specialists, including intensivists, cardiologists, and anesthesiologists.^5^^,^^6^ Currently, no standardized certification or board requirement exists for ECMO training, even within cardiothoracic surgery.^7^ With changes in Accreditation Council for Graduate Medical Education (ACGME) requirements and introduction of new training paradigms for cardiothoracic surgery, general surgery residents often have limited exposure to cardiothoracic surgery early in training. In this context, a surgical ECMO fellowship for residents on dedicated research time offers an opportunity to gain extensive experience in ECMO and mechanical circulatory support (MCS) while simultaneously increasing engagement with the field of cardiothoracic surgery. This manuscript aims to describe the rationale and design of an ECMO/MCS fellowship for general surgery residents, to outline the educational framework and teaching strategies used to deliver progressive clinical and academic training, and to share preliminary experience and lessons learned to guide other programs seeking to implement similar models.

## Preparation

### Program Description

This ECMO/MCS fellowship was established at the University of Colorado Anschutz Medical Center, a large academic medical center with an accredited traditional (5+3) cardiothoracic fellowship program and a recently established 4+3 cardiothoracic training fellowship. The fellowship is intended for general surgery residents with a strong interest in cardiothoracic surgery who have completed 2 or 3 years of general surgery training. The 2-year program is structured so that the first year is primarily clinical while the second year focuses on clinical research.

### Funding Model

Fellowship support is derived from multiple sources, including combined billing revenue (consultation notes, daily management notes, procedures), division or departmental support, and external grants. Fellow coverage reduces the workload on advanced practice providers and traditional cardiothoracic fellows, offering additional justification for resource allocation.

## How I Teach it

### Clinical Responsibilities

The clinical fellow serves as the primary contact for all ECMO consultations and rounds daily on all ECMO and MCS patients in the cardiothoracic intensive care unit (ICU). Fellows collaborate closely with the surgical team, ICU team, ECMO specialists, and consulting services for patient management. They actively assist with cannulations, circuit reconfigurations, decannulations, and operative MCS cases including Impella (Abiomed) placements, left ventricular assist devices, right ventricular assist devices, and heart and lung transplantation. In addition to ECMO cannulations, fellows partake in intraoperative cannulation for planned peripheral cannulations to gain additional practice. Fellows also perform or assist with ICU procedures, such as tracheostomy, chest tube placement, wound care, and device removals. During the first few months of the fellowship, fellows primarily function as assistants during ECMO cannulations and operative MCS procedures. By the second half of the year, fellows frequently serve as primary operators for cannulations, decannulations, and operative MCS procedures under direct attending supervision. In cardiac operating room cases, ECMO fellows typically assist attending surgeons, contributing to exposure, cannulation, and closure. In a few cases in which the case volume exceeds our available traditional fellows, ECMO fellows have had the opportunity to assist with primary cardiac operations and to gain experience with central cannulation. Throughout the fellowship, fellows participate in donor organ procurements, gaining exposure to direct procurement, normothermic regional perfusion, and rapid techniques; during procurements, they work directly with an attending surgeon and gain increased autonomy during the fellowship. Clinical duties are designed to support progressive autonomy and integration with the cardiothoracic team, mirroring an early fellowship experience.

### Research and Program Leadership

The second year of the program emphasizes research in ECMO, MCS, or end-stage cardiopulmonary disease, with reduced clinical duties. Research is primarily focused on clinical outcomes. In addition, fellows can use existing collaborations for increased exposure to translational projects. Fellows take on increased responsibility in program leadership, including contributing to quality improvement initiatives and helping to train the first-year fellow.

### Curriculum and Evaluation

Fellows follow a curriculum designed to gradually increase responsibility and autonomy (Figure). Formal program objectives were created by program leadership, including cardiothoracic surgeons and intensivists, using objectives from similar existing programs as a framework. At the beginning of their training, they work closely with the traditional fellows to gain technical skills and clinical understanding through immersive inclusion in the cardiothoracic surgical team. As skills progress, increased autonomy is granted, with the fellows able to cannulate independently with attending supervision by the end of the first year. Procedural independence is tiered according to competency milestones, similar to ACGME entrustable professional activities. A multicomponent didactic curriculum encompasses both fellowship years and is structured to support progressive learning. Fellows participate in a focused ECMO boot camp at the start of the program to understand ECMO indications and contraindications, ECMO physiology, and how to troubleshoot circuit-related complications. In addition, fellows participate in monthly multidisciplinary ECMO case review and educational conferences, weekly didactics with the traditional cardiothoracic fellows, and asynchronous learning from a provided syllabus.

**Figure.**
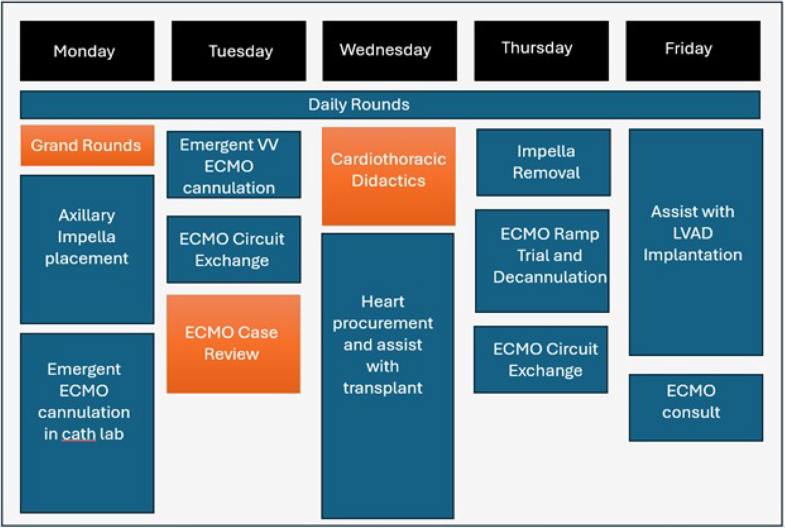
A typical fellowship week integrates intensive care unit management, operative experience, and hands-on participation in heart and lung procurement, extracorporeal membrane oxygenation (ECMO) cannulations, and ramp trials, offering a balanced mix of critical care, operative, and mechanical circulatory support training. (cath lab, catheterization laboratory; LVAD, left ventricular assist device; VV, venovenous.)

Competency is formally assessed in semiannual feedback meetings with the program director. At the completion of the fellowship, fellows are competent in assessing candidacy for, initiating, and managing ECMO and other forms of MCS and have completed search in the fields of end-stage cardiopulmonary disease.

## Comment

### Lessons Learned

Since the implementation of the program, some changes have been made to improve the experience for both fellows and the broader clinical team. Initially, the fellowship operated on a 90/10 model, with the first-year fellow carrying nearly all clinical responsibilities and the second-year fellow minimally involved in direct care. This imbalance underscored the need for shared clinical responsibility to promote sustainability and to avoid burnout, so the decision was made to move to a 75/25 split. Another area of change going forward will be to enhance the on-boarding experience, a need highlighted in early feedback. Planned improvements include an orientation period during which the incoming fellow will work closely with a second-year fellow to gain a solid understanding of the workflow, logistics, and basic tenets of ECMO management; additional simulation training with perfusionists; and creation of an asynchronous didactic curriculum to accelerate early knowledge acquisition. In addition, more rigorous evaluations will be conducted every 6 months, in which fellow competency in a variety of areas will be assessed on a 5-point scale, similar to the ACGME model.

As the program continues to mature and expand, there are plans in place to incorporate a mobile ECMO unit to facilitate ECMO transports and remote cannulations. As part of this, the ECMO fellow would likely play a key role in assisting and participating in remote cannulations with indirect supervision.

### Educational Significance

Overall, preliminary experience demonstrates that the fellowship offers meaningful educational benefits for trainees and may enhance institutional collaboration, although long-term impacts remain to be defined.

From an educational standpoint, the fellowship delivers high-yield clinical experience, technical skill development, and structured mentorship. Fellows gain hands-on exposure to ECMO cannulation, troubleshooting, and perioperative decision-making. By participating in operative cases and ICU management, fellows build a foundation in both the technical and cognitive domains of cardiothoracic surgery. Research and quality improvement activities and mentorship further prepare trainees for academic careers. Fellows serve as a consistent point of contact for ECMO-related issues, which enhances continuity of care and fosters collaboration between the ICU, surgical teams, and perfusion. On an institutional level, the program has improved interdisciplinary communication and continuity of care while reducing the workload for traditional cardiothoracic fellows and advanced practice providers.

Nationwide, most ECMO fellowships are created for intensivists and often require completion of critical care training or general surgery residency before being eligible. Our experiences support the concept that ECMO training early in one’s clinical training offers more value, particularly in terms of exposure to the field of cardiothoracic surgery and mentorship opportunities. Importantly, the fellowship lowers traditional barriers to advanced cardiothoracic operative exposure and mentorship by offering ECMO and MCS training to general surgery residents early in their training pathway. This structure may help democratize access to high-acuity cardiopulmonary care training, particularly at institutions without cardiothoracic surgery residencies, and provides a scaffold for career development regardless of final subspeciality destination.

This pathway may not be useful in all programs as there may be some overlap with integrated cardiothoracic programs (I-6) or heart failure fellowships. However, whereas I-6 may offer similar exposure to ECMO and MCS, most of this occurs later in residency after several years of core rotations. The ECMO/MCS fellowship differs by offering dedicated early exposure during academic development time, before formal cardiothoracic training begins. This structure could potentially complement I-6 programs by fostering early procedural proficiency, research productivity, and mentorship for residents considering a cardiothoracic pathway. In centers with heart failure or transplant fellows, it may be a redundant position or it may be the model can coexist by delineating roles, with ECMO fellows focused on bedside management, cannulation, and perioperative logistics while the advanced fellows direct patient selection and operative strategy.

Beyond training logistics, programs like this may also serve as a template for bridging gaps between surgical residency and subspecialty training. As ECMO and MCS become integral to cardiac and transplant programs, early exposure can improve cross-disciplinary communication, patient safety, and recruitment into cardiothoracic fields. Formalizing these fellowships may also support more widespread increased training in MCS, a growing clinical demand area currently underrepresented in general surgical curricula.

### Conclusion

The creation of a dedicated ECMO/MCS fellowship for general surgery residents demonstrates that meaningful exposure to complex cardiopulmonary support can be delivered early in surgical training. Beyond our experience, this model highlights reproducible teaching strategies—structured boot camps, progressive autonomy, and deliberate integration with multidisciplinary teams—that can be adapted by other high-volume centers. Whereas our initial outcomes are promising, the true value lies in providing a framework that lowers barriers to advanced training and fosters early engagement in cardiothoracic surgery. We hope this experience encourages other programs to adopt and refine similar approaches, ultimately broadening access to high-acuity education and preparing the next generation of cardiothoracic surgeons.
